# Unintentional injury and its prevention in infant: knowledge and self-reported practices of main caregivers

**DOI:** 10.1186/1471-2431-14-132

**Published:** 2014-05-29

**Authors:** Siti Nurkamilla Ramdzan, Su May Liew, Ee Ming Khoo

**Affiliations:** 1 Department of Primary Care Medicine, University of Malaya Primary Care Research Group (UMPCRG), Faculty of Medicine, University of Malaysia, Kuala Lumpur 50603, Malaysia

**Keywords:** Unintentional injury prevention, Infant, Knowledge, Practice

## Abstract

**Background:**

Unintentional injuries are the major cause of morbidity and mortality in infants. Prevention of unintentional injuries has been shown to be effective with education. Understanding the level of knowledge and practices of caregivers in infant safety would be useful to identify gaps for improvement.

**Methods:**

A cross-sectional study was conducted in an urban government health clinic in Malaysia among main caregivers of infants aged 11 to 15 months. Face-to-face interviews were conducted using a semi-structured self-designed questionnaire. Responses to the items were categorised by the percentage of correct answers: poor (<50%), moderate (50% – 70%) and good (>70%).

**Results:**

A total of 403 caregivers participated in the study. Of the 21 items in the questionnaire on knowledge, 19 had good-to-moderate responses and two had poor responses. The two items on knowledge with poor responses were on the use of infant walkers (26.8%) and allowing infants on motorcycles as pillion riders (27.3%). Self-reported practice of infant safety was poor. None of the participants followed all 19 safety practices measured. Eight (42.1%) items on self-reported practices had poor responses. The worst three of these were on the use of baby cots (16.4%), avoiding the use of infant walkers (23.8%) and putting infants to sleep in the supine position (25.6%). Better knowledge was associated with self-reported safety practices in infants (p < 0.05). However, knowledge did not correspond to correct practice, particularly on the use of baby cots, infant walkers and sarong cradles.

**Conclusion:**

Main caregivers’ knowledge on infant safety was good but self-reported practice was poor. Further research in the future is required to identify interventions that target these potentially harmful practices.

## Background

Unintentional injuries in infants have caused significant morbidity and have been a common cause for seeking medical attention [1,2]. In the United States, it is estimated that every one and a half minute, an infant seeks treatment at the emergency department for an unintentional injury [3]. In Singapore, unintentional injuries constitute 7.7% of primary care clinic and emergency department visits. This figure is an underestimation as not all unintentional injuries necessitate medical consultation [4].

In infants, falls are the most common cause of non-fatal injuries [3,5]. Other common causes include ingestion of medication and poison, burns, injuries due to falling objects and motor vehicle accidents. Infants are at high risk of unintentional injuries due to their body size, stage of development, curiosity and inability to anticipate danger [4-6]. Most unintentional injuries occur at home in the presence of caregivers [4]. As infants depend fully on their caregivers, lapses in supervision have been associated with injuries [3].

Unintentional injuries in infants have been shown to be reduced with better knowledge and practices on infant safety. For example, placing the infant in a supine sleep position has been shown to reduce death due to sudden infant death syndrome [7]. Educational intervention to improve knowledge on home safety has been shown to be effective in reducing unintentional injury and in improving safety practices [8].

Hence, it is important to understand the current level of knowledge and practices of infant caregivers to enable us to identify gaps in knowledge and harmful practices for intervention. In this study, we aimed to answer the following research questions: What is the level of knowledge and self-reported practice of caregivers on unintentional injury prevention in infants? Is there any association between knowledge and practice on unintentional injury prevention in infants?

## Methods

A descriptive cross-sectional study was conducted in an urban community health clinic in Petaling Jaya, Malaysia. The inclusion criteria were caregivers who came to the clinic to seek medical attention for their infant and cared for the infant for more than 12 hours a day and who were able to understand English or Malay. The exclusion criterion was caregivers who had obvious cognitive impairment that may affect answering of the questionnaire. The sample size was 403 participants based on a proportion or prevalence of 50% [9], with a non-response rate of 5% based on the response rate of a pilot test done in this study. The proportion or prevalence of 50% is chosen based on the use of baby walkers, which was 55% in Dublin and to cater for maximum variability [10]. Ethics approval was obtained from the University of Malaya Medical Ethics Committee (reference number: 908.16) and the Medical Research Ethics Committee of the Ministry of Health Malaysia (reference number: NMRR-12-667-12346).

The research instrument was a semi-structured self-designed questionnaire. This questionnaire was first developed in English based on the literature on childhood injury and unintentional injury prevention [2-4,11,12]. The literature consists of reports on the most common cause of unintentional injuries worldwide [2-4]. It also includes published suggestions and recommendations on injury prevention in infants and children [11,12]. The questionnaire consisted of 14 items on socio-demographic factors, 21 items on knowledge and 19 items on infant safety practices. The questionnaire was reviewed by one of the researchers (EMK) and three primary care physicians for content validity. It was then translated to Malay, which is the national language, using forward and backward translation by two pairs of independent translators. The translated versions were compared with the English version for semantic and cross-cultural equivalence by two bilingual researchers. Disagreements were discussed and a consensus was reached. A pilot study was conducted for a week. A total of 37 caregivers were approached and they agreed to participate in the pretesting of the questionnaires in both English and Malay for face validity. The questionnaires were found to be easily understood and only minor amendments were made on the order of appearance of the items. The amended questionnaires were subsequently used in the main study.

The study was conducted for 4 months from September 2012 to November 2012 and for May 2013. Caregivers of the infants were approached at the clinic’s registration counter by the interviewer on the day they attended the clinic. Patient information sheet was given and written informed consent was obtained from the participants who agreed to participate at the same time. Face-to-face interviews were then conducted either by the researcher (SNR) or by one of the four enumerators using the questionnaire. The four enumerators were bilingual with a background in science and were trained by the researcher (SNR) on how to conduct the interviews and to complete the questionnaire prior to the study. For each interview, the average time taken was 15 to 20 minutes.

Data were entered and cleaned before analysis using Statistical Package for Social Sciences version 16. The level of knowledge and self-reported practice were reported in descriptive frequencies. They were the correct responses to the items and were further categorised by percentage into poor (<50%), moderate (50% – 70%) and good (>70%) [13]. Associations were tested using Pearson Chi-square test between the responses in knowledge items and the corresponding responses in self-reported practice items. The significance levels for the associations were set at 0.05.

## Results

### Socio-demographic data

A total of 412 caregivers were approached, of which 403 agreed to participate, giving a response rate of 97.8%. Table 1 shows the socio-demographic data of the caregivers. The majority were mothers of the infants (90.6%). Two-thirds of the participants were Malay, and the majority were married and had secondary education or higher. The mean age of the caregivers was 30.1 ± 5.6 years and the mean monthly family income was USD 1251.8 ± 826.7. The mean age of the infants was 13.0 ± 1.4 months, and most infants were born at term with normal birth weight and had no chronic illnesses.

**Table 1:** Socio-demographic background of main caregivers

**Socio demographic factors**	**n = 403[n (%)]**
**Main caregiver**	
Mother	365 (90.6)
Father	35 (8.7)
Grandparent	3 (0.7)
**Gender**	
Male	38 (9.4)
Female	365 (90.6)
**Ethnicity**	
Malay	257 (63.8)
Chinese	81 (20.1)
Indian	30 (7.4)
Others	35 (8.7)
**Mean age ± SD (years) [range]**	30.8 ± 5.6 [19–60]
**Marital status**	
Married	397 (98.5)
Single/divorced/widowed	6 (1.5)
**Highest education level**	
No formal education or primary	20 (5.0)
Secondary school	167 (41.4)
College/diploma	112 (27.8)
University/degree	104 (25.8)
**Monthly mean family income ± SD (USD)**	1251.8 ± 826.7

### Knowledge

**Figure 1:**
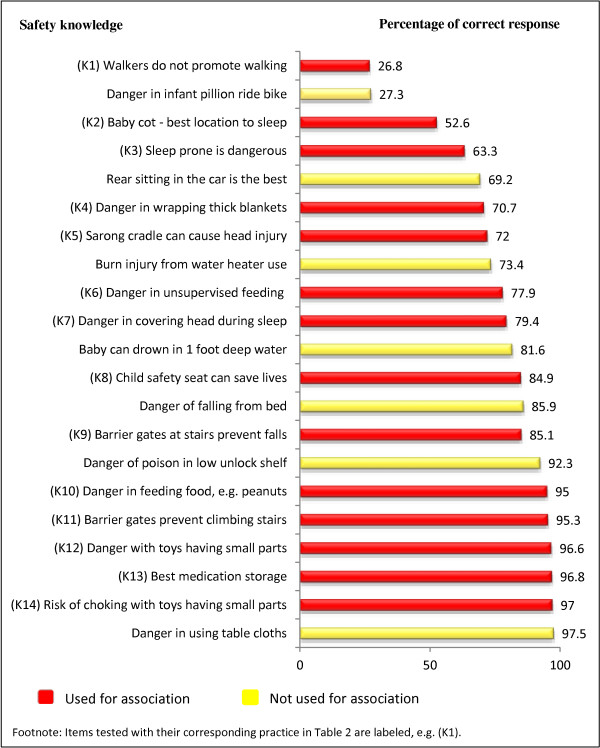
Percentage of correct responses on knowledge statements (N = 403).

Figure 1 shows the percentage of participants with correct answers to the 21 items that assessed knowledge on unintentional injury prevention. Sixteen items had good responses, three items had moderate responses and only two items had poor responses. The two items with poor responses were on the use of infant walkers and the danger to infants to ride on motorcycles as pillion riders. The item that had the highest percentage of correct answers was on the possibility of burns from pulling on table cloths, followed by risk of choking from toys with small parts.

### Self-reported practices

**Figure 2:**
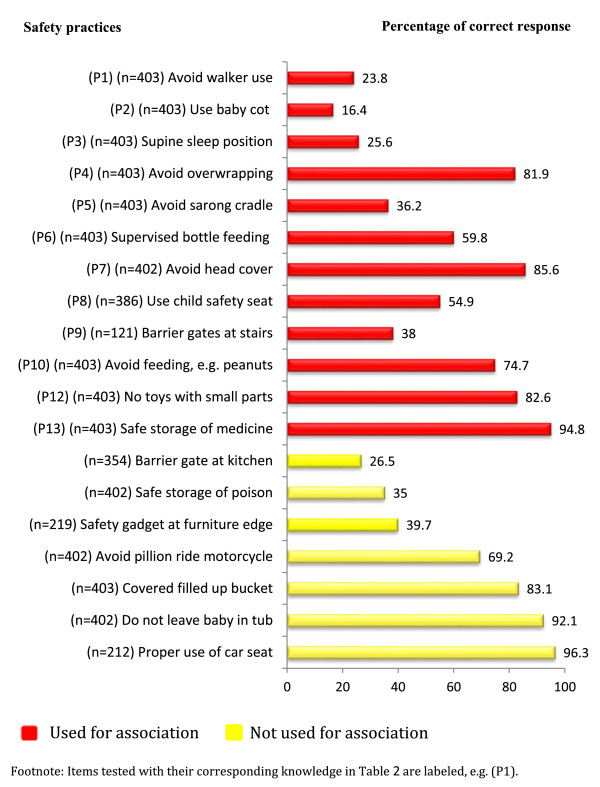
Percentage of correct responses on self-reported practice.

The self-reported practices of caregivers on unintentional injury prevention are shown in Figure 2. The number of caregivers (n) for each item differs; as some of the items were applicable only to some respondents, for example, items on barrier gates at stairs were answered only by caregivers with stairs at their home. None of the participants practised all 19 safety measures. A total of 11 (58%) items on safety practices had moderate-to-good responses and eight had poor responses. Items with the lowest percentage of correct answers were on the use of baby cots (16.4%), use of infant walkers (23.8%) and placing infants in the supine sleep position (25.6%). The item that had the highest correct self-reported practice was on the use of child safety seats (96.3%).

### Association between knowledge and self-reported practices

Table 2 shows the association between knowledge and self-reported practice of main caregivers on unintentional injury prevention. Fourteen of the 21 items on knowledge were tested with their corresponding self-reported practices, for example, K1 with P1. The rest of the items on knowledge were not tested as there were no corresponding self-reported practices. Ten of the 14 tested associations between knowledge and practice were statistically significant. All 10 showed that better safety knowledge was associated with better safety practices. However, in three of these significant associations, more than half of the caregivers who answered the knowledge item correctly did not adhere to its corresponding safety practice. The three associations were on the use of baby cots, infant walkers and sarong cradles.

**Table 2:** Association between knowledge and self-reported practices in unintentional injuries in infants

**Association**	**Knowledge statement**		**Safety practice, n (%)**		**N**	**Χ**^**2**^	**p**
			**Yes**	**No**			
**K1 & P1**	**Walkers do not promote walking**	**Correct**	**44(40.7)**	**64(59.3)**	**108**	**23.27**	<0.001*
		Incorrect	52(17.6)	243(82.4)	295		
**K2 & P2**	**Baby cot** – **best location to sleep**	**Correct**	**54(25.5)**	**158(74.5)**	**212**	**27.02**	<0.001*
		Incorrect	12(6.3)	179(93.7)	191		
K3 & P3	Sleeping prone is dangerous	Correct	199(81.9)	44(18.1)	243	32.64	<0.001*
		Incorrect	89(55.6)	71(44.4)	160		
K4 & P3	Danger in wrapping thick blankets	Correct	260(91.2)	25(8.8)	285	57.28	<0.001*
		Incorrect	70(59.3)	48(40.7)	118		
**K5 & P5**	**Sarong cradles can cause head injury**	**Correct**	**122(42.1)**	**168(57.9)**	**290**	**15.27**	<0.001*
		Incorrect	24(21.2)	89(78.8)	113		
K6 & K6	Danger in unsupervised feeding	Correct	271(86.3)	43(13.7)	314	58.17	<0.001*
		Incorrect	43(48.3)	46(51.7)	89		
K7 & P7	Danger in covering the head during sleep	Correct	291(91.2)	28(8.8)	319	37.38	<0.001*
		Incorrect	54(65.1)	29(34.9)	83		
K8 & P8	Child safety seat can save lives	Correct	193 (58.8)	135(41.2)	328	13.54	<0.001*
		Incorrect	19(32.8)	39(67.2)	58		
K9 & P9	Barrier gates at stairs prevent falls	Correct	38(36.9)	65(63.1)	103	0.371	0.603
		Incorrect	8(41.4)	10(58.6)	18		
K10 & P10	Danger in feeding food (e.g., peanuts)	Correct	296(77.3)	87(22.7)	383	22.73	<0.001*
		Incorrect	6(30.0)	14(70.0)	20		
K11 & P9	Barrier gates prevent climbing stairs	Correct	45(38.5)	72(61.5)	117	0.297	1.000
		Incorrect	1(25.0)	3(75.0)	4		
K12 & P12	Danger with toys having small parts	Correct	326(83.6)	64(16.4)	390	7.76	0.014*
		Incorrect	7(53.8)	6(46.2)	13		
K13 & P13	Best medication storage – top locked shelf	Correct	47(12.1)	340(87.9)	387	0.617	0.433
		Incorrect	3(18.8)	13(81.2)	16		
K14 & P12	Risk of choking with toys having small parts	Correct	325(83.1)	66(16.9)	391	2.20	0.137
		Incorrect	8(66.7)	4(33.3)	12		

*Significant association.

## Discussion

### Main findings

The three main findings of this study are (1) knowledge on infant safety was good as three-quarters of the items had good responses, (2) self-reported practices on infant safety were poor as almost half had poor responses and (3) knowledge on infant safety was not translated to practice in one-third of the tested significant associations. In this study, although better knowledge on safety was associated with better safety practices, there were more caregivers who did not practice safety despite answering the corresponding safety practice in three of the items correctly.

### Knowledge

The knowledge of caregivers on infant safety was good. There were two items that were poorly answered and the worst was on the misconception that ‘infant walkers promote independent walking in infants’. Infant walkers are dangerous and had been shown to be related to unintentional injuries [14]. A study in Singapore had also shown that baby walkers did not accelerate independent walking but led to a delay in motor development [6]. In our study, a total of 73.2% of participants agreed that infant walkers promote independent walking in infants. This is similar in the United States where 72% of caregivers believed that walkers promoted walking and this was one of the reasons for walkers use [15]. Another study in Dublin showed that 75% of parents who used walker believed that walker was good for their infants but only 10% of parents who do not use walker viewed the use of infant walkers as beneficial [9]. Furthermore, 66% of parents who used walker felt that walker was safe, albeit only 5% of parents who do not use walker felt infant walker was safe [9]. Thus, education is important to reduce the rate of infant walker use so that injuries related to it can be prevented.

The other item with poor responses was on the best way for infants to pillion ride on a motorcycle. Most countries do not allow infants to ride on motorcycles but in countries that do, a safety seat must be used [16]. In Malaysia, sidecars and child safety seats for motorcycles are not easily available and there is no legislation prohibiting children from riding on motorcycles. Thus, some caregivers perceived that the safest way to ride was by squeezing the infant between two adult riders. However, it would be safer to use public transport rather than risking injury due to motorcycle accidents.

### Self-reported practices

Overall self-reported safety practices in infants were poor; half of the 19 safety practices had poor responses. The worst self-reported practices were on the use of baby cots, infant walkers and sarong cradles. Very few (13.8%) caregivers used a baby cot, although it has been advocated to prevent falls and sudden infant death syndrome [4,5,17]. A study in the United States showed that the use of baby cots was high and the rate of usage increased with the infants’ age. More than 80% of infants were put to sleep in a baby cot from the age of 9 months [18]. The difference in the rate of use of baby cot may be due to cultural practice as bed sharing is common in Asian countries [19,20]. Another reason could be financial constraints. We have shown that only 25.6% of caregivers placed the baby in a supine sleep position. This rate is much lower than that observed in other studies [18,20]. We did not examine the reason for this practice in this study, but a possible contributing factor could be the older mean age of the infants, which was 13 months. A study in the United States showed that the rates of placing the infant in a supine position reduced in older infants [18].

We found the use of infant walkers was high despite danger associated with their use [14]. Poor knowledge among caregivers on the use of infant walkers was reflected in their practice as a high proportion of them used infant walkers. This was similar to the findings of a study in the United States [15]. Studies in the United Kingdom and Dublin showed that the rate of the use of walker was around 50% [9,21]. Some caregivers used walkers because they felt that walkers were good for their infants [9]. Other reason for the use were previous experience (an older sibling had used it), caretaker’s perception that infants were happy in walkers and having received walkers as a present [9]. Educational counselling to discourage the use of infant walker among parents has been shown to be effective to reduce the use and possession of infant walker [22,23]. Significant reduction in the use of infant walker has been shown to decrease injuries related to it [14,24]. Thus, more education programmes are needed to discourage the use of infant walker.

We also found a high number of caregivers used sarong cradles. There is a lack of studies looking at the use of sarong cradles since the use is unique to the South East Asian region. Sarong cradle is a traditional baby hammock made from cloth and is suspended above the ground anchored with a spring. All injuries sustained with the use of sarong cradle involved the head including serious injuries such as extradural haematoma and skull fracture [25].

The use of sarong cradles and walkers should be discouraged to reduce injuries related to it [6,24,25]. The Canadian government has banned the sale and use of walkers since 2004 [24]. Similarly, the American Association of Pediatrics had also recommended banning the use of infant walkers [14]. Thus, a similar ban on the sarong cradle and infant walker could be effective to reduce their usage in Malaysia.

It was encouraging to find that 96.3% of caregivers used child safety seats properly. This was much higher than the rate of 27.4% found in another local study in 2004 [26]. The higher rate found in this study may be due to an increased awareness of child safety seats from recent national campaigns and a difference in the socio-demographic characteristics of the two study populations.

### Association between knowledge and self-reported practice

This study has shown that better safety knowledge was associated with better safety practices. This indicates that education plays an important role in influencing safety practices of caregivers. A systematic review had shown that education was effective in reducing injuries at home, improved the use of home safety equipment and increased safety practices [8]. Initiatives from public health and clinical authorities are, therefore, recommended to address this issue.

However, it was appalling to note that there were items in which better knowledge was not translated to safe practices. Almost two-thirds of the caregivers answered that a baby cot was the safest sleep location, and yet they did not use a baby cot. Similarly, more than half of the caregivers who used a sarong cradle knew that the use of a sarong cradle could lead to serious head injuries. In addition, more than half of the caregivers used infant walkers despite knowing that their use did not accelerate independent walking. We did not explore the reasons for these discrepancies in knowledge and practice but possible reasons could be financial or cultural [27]. This should be explored in future studies.

## Strengths and limitations

To the authors’ knowledge, there are no previous publications on this topic in Malaysia. This research has added information that filled the research gap. In addition, the response rate of the study was very good (97.8%) and it covered a wide range of topics in unintentional injury prevention in infants.

One of the limitations of this study was that the questionnaire was not validated for reliability. It was self-reported and relied on caregivers’ recall memories on their practice, which could be a potential source of bias. There is also a possibility that respondents might over estimate the number of safe practices to please the researcher since face-to-face interview was used. Nevertheless, it provided an insight on the knowledge and self-reported practices of unintentional injury prevention by caregivers in Malaysia.

## Recommendations

Future qualitative studies are required to explore the barriers of implementing safety practices. Interventions and strategies should target the areas identified in this study where knowledge and practice were poor, particularly in the use of baby cots and baby walkers. An improvement in public knowledge regarding unintentional injury prevention in infants could make a positive impact on the caregivers’ practices.

## Conclusion

The level of knowledge on unintentional injury prevention in infants was good except for the use of baby walkers and motorcycle pillion riding. Overall, self-reported safety practices were poor. Better knowledge was significantly associated with better safety practices. However, there were areas where knowledge did not translate to practice such as in the use of baby cots, sarong cradles and infant walkers. It is uncertain whether these unsafe practices could be due to socio-economic or cultural issues, and further research is required to explore barriers to these safety practices to enable effective intervention.

## Competing interest

The authors declare that they have no competing interests.

## Authors’ contributions

SNR and EMK participated in study design, development and amendment of questionnaire, analysis and interpretation of data and drafting of the manuscript. SML participated in interpretation of data and drafting the manuscript. All the authors read and approved the final manuscript.

## Authors’ information

SNR Lecturer in Department of Primary Care Medicine. SML Associate Professor in Department of Primary Care Medicine. EMK Professor in Department of Primary Care Medicine.

## Pre-publication history

The pre-publication history for this paper can be accessed here:

http://www.biomedcentral.com/1471-2431/14/132/prepub
